# G protein selectivity profile of GPR56/ADGRG1 and its effect on downstream effectors

**DOI:** 10.21203/rs.3.rs-4869264/v1

**Published:** 2024-09-05

**Authors:** Raida Jallouli, Ana Lilia Moreno Salinas, Andréanne Laniel, Brian Holleran, Charlotte Avet, Joan Jacob, Trang Hoang, Christine Lavoie, Kendra S Carmon, Michel Bouvier, Richard Leduc

**Affiliations:** Université de Sherbrooke: Universite de Sherbrooke; Université de Sherbrooke: Universite de Sherbrooke; Université de Sherbrooke: Universite de Sherbrooke; Université de Sherbrooke: Universite de Sherbrooke; University of Montreal: Universite de Montreal; The University of Texas MD Anderson Cancer Center; University of Montreal: Universite de Montreal; University of Sherbrooke: Universite de Sherbrooke; The University of Texas MD Anderson Cancer Center; Université de Montréal: Universite de Montreal; University of Sherbrooke: Universite de Sherbrooke

**Keywords:** G protein-coupled receptor, GPR56, BRET, RhoGEF, signaling, trafficking

## Abstract

GPR56, an adhesion G-protein coupled receptor (aGPCRs) with constitutive and ligand-promoted activity, is involved in many physiological and pathological processes. Whether the receptor’s constitutive or ligand-promoted activation occur through the same molecular mechanism, and whether different activation modes lead to functional selectivity between G proteins is unknown. Here we show that GPR56 constitutively activates both G12 and G13. Unlike constitutive activation and activation with 3-a-acetoxydihydrodeoxygedunin (3αDOG), stimulation with an antibody, 10C7, directed against GPR56’s extracellular domain (ECD) led to an activation that favors G13 over G12. An autoproteolytically deficient mutant, GPR56-T383A, was also activated by 10C7 indicating that the tethered agonist (TA) exposed through autocatalytic cleavage, is not required for this activation modality. In contrast, this proteolysis-resistant mutant could not be activated by 3αDOG indicating different modes of activation by the two ligands. We show that an N-terminal truncated GPR56 construct (GPR56-Δ1–385) is devoid of constitutive activity but was activated by 3αDOG. Similarly to 3αDOG, 10C7 promoted the recruitment of b-arrestin-2 but GPR56 internalization was β-arrestin independent. Despite the slow activation mode of 10C7 that favors G13 over G12, it efficiently activated the downstream Rho pathway in BT-20 breast cancer cells. These data show that different GPR56 ligands have different modes of activation yielding differential G protein selectivity but converging on the activation of the Rho pathway both in heterologous expressions system and in cancer cells endogenously expressing the receptor. 10C7 is therefore an interesting tool to study both the processes underlying GPR56 activity and its role in cancer cells.

## Introduction

G protein-coupled receptors (GPCRs) form the largest protein superfamily in the human genome. They regulate a broad spectrum of processes both at the physiological and pathophysiological levels [1–3]. Adhesion GPCRs (aGPCR) represent the second largest family of GPCRs in humans with 33 members [4]. One of the most studied members of the aGPCR family, GPR56/ADGRG1, is expressed in many tissues, including pancreatic islets, the thyroid gland and the kidney as well as in lymphoid and brain tissues [5, 6]. Recently, it has been associated with a variety of human disorders ranging from neurological and psychiatric disorders to many cancer types [7–10].

Like all aGPCRs, GPR56 consists of topology-based and cleavage-based compartmentations. The topology-based compartmentation consists of a three-partite structure: 1) the extracellular domain (ECD), which is thought to be responsible for cell-cell and cell-matrix communications due to the presence of conserved adhesion motifs [11], contains an N-terminal PLL (Pentraxin and Laminin/neurexin/sex-hormone (LNS)-binding-globulin-Like) domain that is unique to GPR56 [12], a GAIN (GPCR Autoproteolysis INducing) domain harboring a GPCR Proteolysis Site (GPS), 2) the seven transmembrane domain structure with extracellular and cytoplasmic loops (7TMs) and 3) the intracellular domain (ICD) which is the receptor’s cytoplasmic tail. The cleavage-based compartmentation consists of a two-partite structure: 1) the NTF contains the adhesion domains and most of the GAIN domain and 2) the CTF comprises the C-terminal portion of the GAIN domain known as the “Stachel” or “stalk” with the first approximative seven amino acids comprising a putative tethered agonist (TA) [13–15] similar to what has been described for the protease-activated receptors [16, 17] as well as the 7TM portion of the receptor and the cytoplasmic carboxyl tail. Both the NTF and CTF remain non-covalently associated and exposed to the cell surface.

Three potential mechanisms have been proposed to explain the activation of aGPCRs at the molecular level: 1) The tunable model where ligand binding potentially induces conformational changes between the NTF and the CTF thereby triggering receptor signaling that is independent of the exposure of the TA [18, 19]; 2) the shedding model, or the TA dependent model, in which the binding of ligands enables the freed TA to interact with the CTF and promote receptor signaling [20, 21]]; and 3) the mechano-dependent model where mechanic-based stimulation triggers allosteric changes in the GAIN domain enabling the engagement of the TA with the 7TM without dissociation of the NTF [22]. Signaling studies have shown that GPR56 exhibits some level of constitutive activity and promote serum response element (SRE) and serum response factor response element (SRF-RE) through RhoA signaling [19, 23]. Besides this constitutive activity, it was proposed that Collagen-III [6, 24] and Transglutaminase 2 (TG2) [25, 26] present in the extracellular matrix (ECM) act as two GPR56 interaction partners by linking with the receptor’s PLL domain. While the interaction with Collagen-III results in activation of the RhoA pathway, the molecular mechanism resulting from the interaction with TG2 has not been fully elucidated. In addition to these natural agonists, peptides that mimic GPR56’s TA have been shown to promote GPR56-mediated RhoA-serum response element, SRF/SRE signaling [20, 21]. A small synthetic molecule, 3-α-acetoxydihydrodeoxygedunin (3-α-DOG), was also shown to act as a partial agonist for GPR56 as measured by the SRE-luciferase assay [27]. Antibodies raised against different regions of the GPR56 ECD, especially the PLL and the GAIN domain, behave as agonists but their precise signaling modalities are unknown [12, 28, 29]. Recently, a monoclonal antibody (mAb), 10C7, directed against GPR56’s GAIN domain, was shown to potentiate RhoA-SRF signaling [23, 30].

GPR56 signaling studies have mostly been restricted to the accumulation of cAMP [31] or the measure of specific transcriptional reporter genes [19, 20, 30] (i.e., SRE, SRF-RE, NFAT). Given that many GPCR can engage multiple signaling pathways we wanted to determine whether the different modes of GPR56 activation (constitutive vs ligand-mediated) lead to the engagement of different signaling pathways by using a combination of GPR56 ligands, GPR56 mutants and bioluminescence resonance energy transfer (BRET) assays [32–34]. Thus, the present study aimed at: 1) identifying the G protein and b-arrestin repertoire engaged by GPR56, 2) establishing whether the same signaling effectors are engaged by the constitutively active receptor vs the receptor activated by different agonists, 3) unraveling which of the molecular activation models proposed for GPR56 activation are responsible for its constitutive and agonist-mediated responses. Our data reveal that 1) GPR56 constitutively couples to Gas, Ga12 and Ga13, 2) the constitutive activity profile of GPR56 is distinct from the one promoted by the antibody 10C7, which favors GPR56 coupling to Ga13, but similar to the one promoted by the agonist 3-a-DOG, 3) the constitutive activity of GPR56 toward Gα12/13 does not require the autoproteolytic cleavage of the ECD and is thus independent of an exposed TA, 4) the 10C7 antibody is a GPR56 agonist that activates both cleaved and noncleaved forms of the receptor, indicating that as for the constitutive activity, the exposed TA is dispensable for its agonist activity. This contrasts with the agonist activity of the small molecule 3αDOG that requires ECD autoproteolysis for its ability to promote Ga12/13 activation, 5) GPR56 is internalized in a β-arrestin independent manner and 6) despite the distinct signaling profile of 10C7 we found that it stimulated endogenous GPR56, which resulted in RhoA activation in breast cancer cells (BT20) confirming its agonistic activity in a pathophysiological context.

## Methods

### Reagents

Coelenterazine 400 A was from GoldBio, Prolume Purple and nanofuel solvent were from Nanolight technology. 3αDOG was purchased from Microsource Discovery. The pGL4.34 [luc2P/SRF-RE/Hygro] and pGL4.75 [hRluc/CMV] vectors were purchased from Promega. hGPR56-WT cDNA was from R&D system (Catalog Number: RDC0140), corresponding to variant 1 derived full length 693 aa receptor, and cloned between KpnI and XbaI sites of the pcDNA3.1/Amp(+) vector. Δ1–385 was generated using the following primers: FW 5’- GTCTCCGGATTCGAATTCAGATGGCAGTGCTGATGGTCT-3’ and REV 5’GTCATGGTACCGGCGCTTCCGATGCGGCTGGACGACGAGGT-3’. T383A mutant was generated as previously described [23], the T383/F385/M389-A triple mutant was generated using the primers FW 5’ – GGCCTACGCTGCAGTGCTGGCGGTCTCCTCGG-3’and REV 5’-CCGAGGAGACCGCCAGCACTGCAGCGTAGGCC. Sequences encoding Gαs-117-RlucII, Gαq-118-RlucII, Gαi1-91-RlucII, Gαi2-loop-RlucII, Gαi3-loop-RlucII, GαoA-99-RlucII, Gαz-94-RlucII, Gα12–136-RlucII, Gα13–130-RlucII, β-arrestin2-RlucII, PKN-RBD-RlucII, GFP10-Gγl,rGFP-CAAX, p115-RhoGEF-RlucII and PDZ-RhoGEF-RlucII were previously described [33–37]. Full-length, untagged Gβ1 (Cat. #GNB0100000), GNA12 (Gα12) (Cat. # GNA120EI00) and GNA13 (Gα13) (Cat. # GNA130EI00) were purchased from the cDNA resource center. Anti-GPR56 mAb 10C7 was generated and sequenced as previously described [23]. C-terminal anti-GPR56 antibody (ABS1028) was from Sigma-Aldrich. Anti-Flag Ab (F7425) and Anti-myc Ab (06–549) were from Sigma-Aldrich. Horseradish peroxidase-conjugated goat anti-human IgG secondary antibody, Alexa-Fluor 594 donkey anti-rabbit and Alexa-Fluor 594 donkey anti-mouse conjugated secondary antibodies were obtained from Thermofisher Scientific. GAPDH (D16H11) rabbit mAb (HRP Conjugate), β-arrestin1/2 Rabbit mAb (D24H9) and anti-rabbit IgG (7074) (HRP linked) were from Cell Signaling. Dulbecco’s modified Eagle’s medium (DMEM), Eagle’s minimal essential medium (EMEM), Phosphate Buffered saline (PBS), fetal bovine serum (FBS), penicillin-streptomycin-glutamine (PSG), and (4-(2-hydroxyethyl)-1-piperazineethanesulfonic acid) (HEPES) were purchased from Wisent (St-Bruno, QC, Canada). TryPLE Express Enzyme with phenol red was obtained from Gibco (Gaithersburg, MD, USA). Opti-MEM was purchased from Invitrogen (Burlington, ON, Canada). Polyethylenimine (PEI) was acquired from Polyscience, Lipofectamine 2000 was from ThermoFisher and Fugene 6 was purchased from Promega. The cell surface biotinylation kit was from Thermofisher (A44390) and the RhoA G-LISA activation assay kit was purchased from Cytoskeleton (BK124).

### Cell culture and transfections

HEK293SL cell line was a kind gift from S. Laporte (McGill University, Montreal, Quebec, Canada), hereafter referred to as HEK293 cells. HEK 293T cells devoid of functional Gα12 and Gα13 proteins (ΔGα12/13 cells) were kindly provided by A. Inoue (Tohoku University, Sendai, Miyagi, Japan). Human breast cancer cells (BT-20) were purchased from ATCC. Myc-GPR56 stable HEK293T cell line were generated as previously described [23]. All cell lines were cultured in DMEM medium supplemented with 10% FBS and 1% antibiotics (100 U/mL penicillin and 100 μg/mL streptomycin, PS) except for the BT-20 cell line which was grown in EMEM medium supplemented with 10% FBS and 1% PS. Cells were cultured at 37°C in a 5% CO_2_ humidified incubator. All transient transfections were performed with PEI reagent.

Before BRET signals were recorded, cells at 3.5×10^5^ cells/mL were transfected with 1 μg of total DNA diluted in 100 μL Opti-MEM (adjusted with pcDNA3.1; Genescript) using a 3:1 ratio of linear PEI (1 mg/mL) per μg DNA. For LPAR1 a gelatin solution (1%; Sigma-Aldrich) was used to stabilize DNA/PEI transfection mixes. Cells were then directly seeded (3,5×10^4^ cells/well) in white opaque 96-well microplates (Perkin Elmer) and incubated for 48h.

The G-protein constitutive activation profile was elucidated with G protein-based BRET^2^ biosensors based on the separation of human Gα-RlucII and GFP10-Gγ1 in the presence of Gβ1. Biosensor expression vectors were used as per the following scheme: 40ng of plasmid encoding RlucII-Gα (Gα12, 13, q, i1, i2, i3, z, oA, and s), 250 ng of plasmids encoding Gβ1 and 250 ng of GFP10-Gγ1 in the presence or absence of 125 ng of plasmid encoding GPR56-WT. For the effector membrane translocation assays (GEMTA), the sequence encoding the Gα12/13 binding domain of the human p115-RhoGEF (residues 1–244) [33] and PDZ-RhoGEF (residues: 281–483) [34] was tagged with RlucII. These sensors were used to monitor GPR56 constitutive and induced activation of Gα12/13 proteins, in ΔGα12/13 cells, by following their recruitment to the plasma membrane where the rGFP-CAAX (a polybasic sequence with the prenylated CAAX box of the GTPase first identified in Kirsten Rat Sarcoma virus [KRAS]) was anchored, in presence or no of either Gα12 or Gα13 with increasing amounts of WT or mutant forms of GPR56. When using p115-RhoGEF biosensor, 10 ng of plasmid encoding p115-RhoGEF-RlucII and 300 ng of plasmid encoding rGFP-CAAX, supplemented or no with 40 ng of plasmid encoding Gα12 in the presence of different amounts of GPR56-WT or mutants were transfected. For the PDZ-RhoGEF biosensor, cells were transfected with 3 ng of plasmid encoding PDZ-RhoGEF-RlucII and 200 ng of plasmid encoding rGFP-CAAX, supplemented or not with 40 ng of plasmid encoding Gα13 and different amounts of GPR56. For the Rho sensor, HEK 293 cells were transfected with 120 ng of plasmid encoding PKN-RBD-RlucII, 480 ng of plasmid encoding rGFP-CAAX and 125 ng of plasmid encoding GPR56-WT were transfected.

β-arrestins recruitment to the plasma membrane, after 10C7 or 3αDOG GPR56-WT stimulation, was determined by monitoring the BRET signal between 40 ng of plasmid encoding β-arrestin1 or 2-RlucII and 250 ng of plasmid encoding rGFP-CAAX.

### BRET measurements

The day of the experiment, medium was removed and replaced by the BRET buffer (in mM: 10 HEPES, 1 CaCl2, 0.5 MgCl2, 4.2 KCl, 146 NaCl, 5.5 glucose, pH 7.4) and cells were incubated at 37°C for 1h. For all G protein-based biosensors, the BRET^2^ ratio was normalized with the one calculated in the absence of a receptor. For concentration-response curves with GEMTA biosensors, cells expressing GPR56-WT were stimulated with increasing concentrations of 10C7 for 20 min at 37°C and cells expressing LPAR1 were stimulated with increasing concentrations of oleoyl-LPA for 10 min before BRET signal measurement. To measure the synergy between 10C7 and 3αDOG, cells expressing 125 ng of plasmid encoding GPR56-WT were stimulated with increasing concentrations of 3;1DOG alone or in the presence of a constant concentration of 10C7 (10 nM for p115-RlucII or 5nM for PDZ-RlucII) for 20 min at 37°C. Coelenterazine 400a (final concentrations of 5 μM) was added 5 min before BRET measurement. For kinetic measurement of GPR56-WT and GPR56-T383A mutant activation by 10C7 or 3αDOG, and GPR56-Δ1–385 mutant with 3αDOG, basal BRET was measured during 100 sec before stimulation with either vehicle (buffer or DMSO 0.1%) or 20 nM of 10C7 or 10 μM of 3αDOG in the presence of 2 μM of Prolume purple coelenterazine and BRET signal was recorded each 30 sec during at least 1500 sec. To measure the effect of 10C7 on the GPR56 triple mutant signaling, cells expressing 125 ng of plasmids encoding GPR56-WT, GPR56-T383A, GPR56-Δ1–385 or GPR56 T383/F385/M389-A together with GEMTA biosensors and the corresponding Gα protein were stimulated with 20 nM of 10C7 for 30 min at 37°C, and coelenterazine 400a (final concentrations of 5 μM) was added 5 min before BRET measurement. Plates were read on the Berthold TriStar2 LB 942 Multimode Reader (Berthold, Bad Wildbad, Germany) with the energy donor filter (410 nm) for RlucII and energy acceptor filter (515 nm) for GFP10 and rGFP-CAAX. The BRET signal (BRET^2^) was determined by calculating the ratio of the light intensity emitted by the acceptor over the light intensity emitted by the donor. For synergy experiment between 10C7 and 3αDOG, the BRET^2^ ratio was normalized with respect to the signal obtained after stimulating GPR56 with the lowest concentration of 3αDOG alone.

### Data analysis

For BRET experiments, data were analyzed in GraphPad Prism 9 software and each result is represented as the mean ± SD of at least three independent experiments performed in triplicate. Statistical analyses were performed using GraphPad Prism 9 software and the specific test used to determine whether differences are statistically significant are described in the figure legend. Significance was determined as p < 0.05.

### Luciferase reporter assay

For the SRF-RE activity, a dual luciferase reporter assay was used. HEK293 cells were transiently transfected in 96-well plates (3.5×10^4^ cells/well) with pGL4.34 (SRF-RE) driving the transcription of Firefly luciferase reporter gene upon SRF activation, pGL4.75, a *Renilla* luciferase reporter gene used as an internal control to normalize transfection efficiency and variant amounts of receptor DNA. After six hours, cells were starved for 16h at 37°C. For 10C7-induced SRF-RE activation, serial dilutions of the mAb were added to cells and incubated for 20 min at 37°C. Firefly and *Renilla* luciferase activities were measured using the Dual-Glo^®^ luciferase Assay System (Promega) according to the manufacturer’s instructions.

### cAMP measurements

We used the HTRF cAMP Gs Dynamic kit from Revvity Health Sciences Canada, Inc (Mississauga, Ontario, Canada) to measure cyclic adenosine monophosphate (cAMP) levels. 48 hours after transfection, cells were washed with PBS at room temperature, then trypsinized and distributed at 5 000 cells/well (5 μl) in a white 384-well plate in stimulation buffer (10 mM Hepes, 1 mM CaCl_2_, 0.5 mM MgCl_2_, 4.2 mM KCl, 146 mM NaCl, 5.5 mM glucose, 0.5 mM IBMX, pH 7.4). Necessary dilutions of each ligand (2X) in addition to forskolin (10 uM) were prepared in stimulation buffer and cells were stimulated at 37°C for 30 min with indicated ligand (5 uL). Cells were then lysed with the lysis buffer containing 5 μl of cAMP coupled to the d2 dye. After addition of 5 μl of anti-cAMP cryptate terbium conjugate, cells were incubated for 3 h at room temperature under agitation. FRET signal was measured using a TECAN M1000 fluorescence plate reader (TECAN, Austria). Results were normalized to the forskolin response for each condition.

### Western Blot analysis

Cells were seeded in six-well plate (5 × 10^5^ cells/well). After 24h, cells were transfected with the indicated amounts of DNA. Twenty-four hours later, cells were starved overnight in serum-free medium. The following day, cells were washed with ice-cold PBS buffer and proteins were extracted using a buffer containing 50 mM Tris (pH 7,4) 150 mM NaCl, 5 mM EDTA and 1% Triton X-100. Cell lysates were centrifuged at 15,000 rpm for 20 minutes at 4°C. Equal amounts of proteins were separated by SDS-PAGE and transferred onto nitrocellulose membrane using iBlotTM 2 Transfer stacks (ThermoFisher Scientific). Proteins were detected using specific antibodies targeting the protein of interest. HRP-labeled secondary antibodies were utilized for detection with the standard ECL protocol (BioRad). Western blots shown are representative of three independent experiments. Relative densitometry analysis on protein bands was performed using Bio1D software.

### Cell surface ELISA

HEK293 cells were plated into 6-well plates and transiently transfected with cDNA encoding GPR56-WT, GPR56-T383A or GPR56 T383/F385/M389-A. Cells were transfected with a constant amount of total DNA (1 μg/well), with a ratio of 3:1 PEI: DNA. Twenty-four hours after transfection the cells were transferred to 24-well plates (2.5×10^5^ cell/well). Forty-eight hours after transfection, cells were washed with PBS, fixed (formaldehyde 3.7%) and then incubated in blocking buffer (TBS 1X + 5% milk) for 30 min at room temperature with shaking. Blocking buffer was then removed and replaced with fresh blocking buffer supplemented with primary antibody directed against ECD: 10C7 (1:15) and incubated for 60 min at room temperature with shaking. 10C7 was then removed and cells were washed twice with TBS1X and incubated for 30 min with diluted goat anti-human IgG secondary antibody (1:15000). Cells were then washed three times with TBS 1X incubated with Tetramethylbenzidine liquid substrate (TMB, Sigma T0440) until a color change was observed (5 min). Reactions were stopped by the addition of 2 N hydrochloric acid, and absorbance at 450nm determined with a Berthold TriStar2 LB 942 Multimode Reader. A statistical comparison of activation levels was performed using an unpaired student’s t test.

### Cell Surface Biotinylation

HEK293 cells were transfected with empty vector DNA or GPR56-WT, GPR56-T383A and GPR56-Δ1–385. At 24 h post-transfection, cells were placed on ice and washed with ice-cold PBS. Cells were then incubated with sulfo-NHS-SS-Biotin on ice for 30 min and then washed twice with ice-cold TBS. Cells were then scrapped, centrifuged and resuspended in 500μL of lysis buffer for 30 min at 4°C. Cell debris was cleared by centrifugation and equal amounts of protein (BCA Protein Assay kit) were incubated with neutravidin agarose beads for 30 min at 4°C. Beads were washed three times with lysis buffer and labelled proteins were eluted with 200 μL Elution buffer and 25μL DTT stock solution for 30 min at RT with end-over-end mixing on the rotator. Biotinylated proteins were detected via western blot, as described above, using the 10C7 as a primary antibody and goat anti-human IgG as secondary antibody.

### RhoA ELISA activation assay

To determine the amount of activated RhoA in BT-20 cells following 10C7 stimulation, an ELISA-based assay quantifying the amount of the active GTP-bound form of RhoA was used according to the manufacturer’s protocol (RhoA G-LISA activation assay kit, Cytoskeleton). Briefly, BT-20 cells were grown to 60% confluency and starved in serum-free EMEM overnight. The following day, cells were treated or not with 200 nM 10C7 (15 min) or a RhoA activator I (Calpeptin, Cytoskeleton) (10 min) used as a positive control. Cells were then washed with ice-cold PBS, lysed, clarified by centrifugation, and snap frozen. For each GLISA experiment, 20 μL of 0,5 mg/mL duplicates for each condition were used and the signal was read by measuring absorbance at 490 nm using a microplate spectrophotometer. Each condition was repeated three times, and a statistical comparison of activation levels was performed using a paired student’s t test.

### Immunofluorescence and confocal microscopy

siRNAs against β-arrestin1, β-arrestin2, clathrin and the non-targeting control siRNA were purchased from Dharmacon (catalog no. L-007292-00-0005, L-011971-00-0005, M-004001-00-0005 and D-001810-10-05, respectively). GPR56 stable cell line seeded on poly-lysine coated coverslips at a density of 10^5^ cells were first transfected with siRNAs (final concentration of 100 nM) using lipofectamine 2000 and incubated for 72h before analysis. For experiments using the AT1 receptor, siRNA-transfected cells were transfected the following day with FLAG-AT1 using Fugene 6 according to the manufacturer’s instructions. GPR56 and AT1 cell surface labeling assays were performed at 4°C using 1:500 diluted anti-Myc Rabbit Ab or 1:500 diluted anti-FLAG mouse Ab, respectively, for 1h. Cells were first treated with 15 μg/mL 10C7 or 1 μM Angiotensin II at 37°C for 30 min to trigger internalization. Cells were then fixed in 4% paraformaldehyde, quenched 10 min with 50 mM NH_4_Cl, permeabilized 10 min with 0.1% Triton X-100 and blocked 30 min with 10% FBS. Coverslips were incubated 1 h with Alexa Fluor 594-conjugated donkey anti-rabbit or Alexa-Fluor 594-conjugated donkey anti-mouse secondary antibodies at room temperature. Cells were then washed three times with 1% goat serum and the coverslips were mounted on glass slides using Prolong Glass Antifade Mountant (Invitrogen, catalog no. P36981). Nuclei were counterstained with Hoechst solution. Images were acquired with an LSM Olympus FV1000 spectral confocal microscope with a 60x oil immersion objective. Olympus FluoView version 4.2b was used to analyze images.

## Results

### G-protein constitutive coupling profile of GPR56

**Figure 1:**
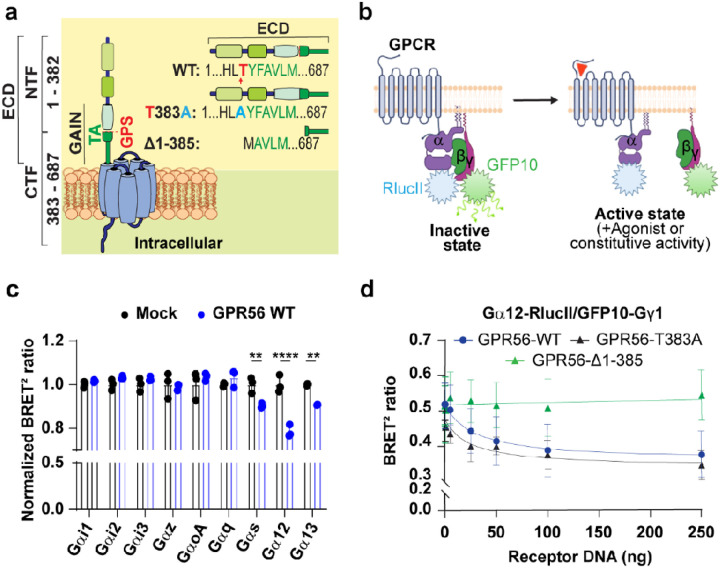
BRET G protein coupling profile of GPR56-WT and Mutants. **a** Schematic representation of GPR56-WT, autocleavage deficient mutant GPR56-T383A and GPR56-Δ1–385 mutant in which 3 amino acids were deleted from the N terminus of the Tethered Agonist (TA). GPS is indicated by a red arrow and Tethered Agonist sequence is TYFAVLM in which T (red) is the GPS P1’ residue. Abbreviations are, ECD, Extracellular domain; NTF, N-terminal fragment; CTF, C-terminal fragment, GAIN, GPCR Autoproteolysis Inducing domain; TA, Tethered Agonist, GPS, GPCR Proteolysis Site; ICD, Intracellular Domain. **b** Schematic diagram of the G protein BRET biosensor (GABY) which measures the activation of G protein resulting in distancing of RlucII from GFP10 and a decrease of BRET signal, created with BioRender.com. **c** BRET-based G protein activation profile in the presence of 125ng GPR56-WT cDNA represented by the BRET ratio normalized with that measured in the absence of GPR56-WT using empty vector (Mock). **d** Representative GPR56-WT and mutants BRET^2^ ratio curves with the Ga12 BRET sensor in relation to increasing receptor DNA concentrations (0–250ng). Data are represented as the mean values of at least three independent experiments of three replicates each (mean ±SD).

To assess the constitutive G-protein coupling activity of GPR56, we first performed parallel studies using three forms of the receptor: 1) GPR56-WT, 2) the GPS cleavage deficient mutant where we introduced a point mutation (GPR56-T383A) that abolishes the receptor’s autocleavage site [19, 23] and 3) GPR56-α1–385, lacking the N-terminal fragment and three amino acids from the N-terminal extremity of the TA, thus inactivating the TA (Fig. 1a).

We first measured the ability of GPR56-WT to engage G proteins by virtue of its constitutive activity using G protein BRET-based biosensors where the Gα and Gγ subunits are fused to RlucII and GFP10 [35] respectively (Fig. 1b). Upon activation of the receptor a spatial distancing between Ga-RlucII and Gbg-GFP10 occurs leading to a decrease in the BRET signal of this Gabg-based sensor (Gaby). We monitored the response upon heterologous co-expression of GPR56-WT and specific Ga subunits in HEK293 cells. Constitutive coupling to Gαs, Gα12 and Gα13 was observed upon heterologous expression of increasing amounts of GPR56 as revealed by the BRET decrease (Fig. 1c). No such constitutive engagement was detected for Gαq, Gαi1, Gαi2, Gαi3, Gαz and Gαo indicating a selective coupling for the Gs and G12/13 pathways. To elucidate the molecular mechanisms underlying GPR56 constitutive activity we took advantage of the more robust signal observed for Gα12. For this purpose, we expressed forms of GPR56 that lack the GPS cleavage site (GPR56-T383A) or lack a large part of the ECD resulting in a truncated TA. The constitutive coupling capacity was assessed by monitoring the impact of expressing increasing concentration of the various GPR56 constructs on the Ga12 biosensor activity (Fig. 1d). Compared to GPR56-WT, the uncleavable GPR56-T383A, was well expressed (Fig S1a) and showed a 1.5-fold increase in cell surface expression (Fig S1 b,c) and exhibited an important level of constitutive engagement towards Ga12 (Fig. 1d). In contrast, no constitutive Gα12 coupling was observed with the GPR56-Δ1–385 mutant, consistent with a role of the ECD domain in constitutive activation. Although its surface expression could not be verified using the antibody recognizing the N-terminus since it is deleted, GPR56-Δ1–385 total expression level was determined using an anti-GPR56 C-terminal antibody and showed an expression level comparable to the WT form (Fig S1d). Additionally, the fact that this mutant could be activated by 3aDOG lends additional support to its presence at the cell surface. It follows that although the ECD is important for GPR56 constitutive activity, it is independent of its cleavage status indicating that the spontaneous engagement of Gα12 does not involve the exposition of the TA.

### GPR56 constitutive activity toward Gα12/13 monitored by BRET-based effector membrane translocation assays

**Figure 2:**
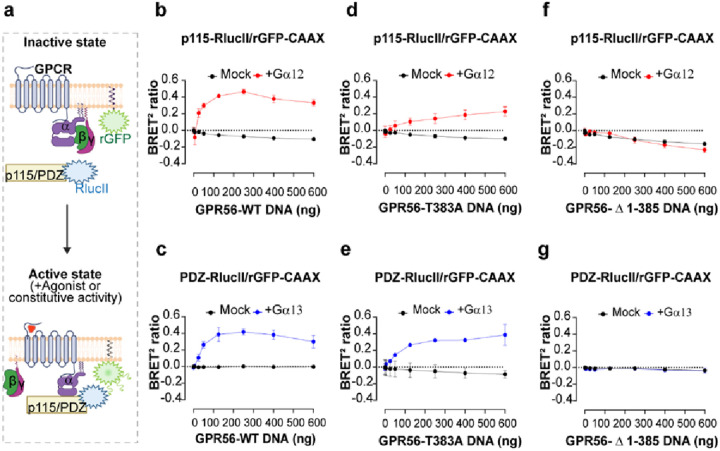
BRET-based effector membrane translocation assay to assess GPR56-WT and mutants’ constitutive activity. **a** Schematic representation of the GEMTA ebBRET-based biosensors to monitor Ga12/13 protein activation. Upon constitutive or agonist stimulation, activated Ga12 or Gα13 subunits recruit their selective downstream effector p115-RhoGEF-RlucII or PDZ-RhoGEF-RlucII to the plasma membrane (PM) where rGFP-CAAX is anchored, leading to an increase of ebBRET, created with BioRender.com. **b**, **d**, **f** Constitutive activation of Gα12 protein by GPR56-WT and mutants, reflected by the selective recruitment of the p115-RhoGEF effector to the PM. **c**, **e**, **g** Constitutive activation of Gα13 proteins by GPR56-WT and mutants, reflected by the selective recruitment of the PDZ-RhoGEF effector to the PM. HEK293 ΔGα12/13 cells were transiently transfected with an increasing amount of receptor DNA and p115-RhoGEF-RlucII/rGFP-CAAX or PDZ-RhoGEF-RlucII/rGFP-CAAX biosensors, supplemented or not with the Gα12 or Gα13 subunits, respectively. Data are represented as the mean ± SD of three biological replicates.

To further confirm that the constitutive engagement of Gα12 revealed by the Gaby sensors above resulted in the activation of the Gα12/13 family members, we took advantage of the enhanced bystander BRET (ebBRET)-based G protein effector membrane translocation assays (GEMTA) that directly monitors the activity of G proteins by measuring the recruitment of a fragment of their downstream effectors [34]. To characterize the constitutive activity of GPR56 toward G12 and G13 we used p115-RhoGEF-RlucII and PDZ-RhoGEF-RlucII as energy donors coupled to rGFP-CAAX as an energy acceptor. Given that p115-RhoGEF yields a more robust signal for Gα12 than Gα13 and that the contrary is true for PDZ-RhoGEF (Fig.S2), p115-RhoGEF-RlucII was used to monitor G12 activation whereas PDZ-RhoGEF-RlucII was used for G13. The ability of GPR56-WT, GPR56-T383A or GPR56-Δ1–385 to constitutively activate G12 or G13 was assessed in Gα12/13-KO HEK293 cells (ΔGα12/13 cells) in which either Gα12 or Gα13 were heterologously expressed individually (Fig. 2).

A receptor concentration-dependent constitutive recruitment of PDZ-RhoGEF and/or p115-RhoGEF was detected for the GPR56-WT following reintroduction of Gα13 or Gα12, respectively. Interestingly, the dynamic range for the BRET ratio was similar using the two biosensors reaching a maximum of 0,44 ± 0,02 at 250 ng of transfected GPR56-WT DNA (Fig. 2b, c). The selectivity of the signal is confirmed by the fact that no increase in ebBRET between rGFP-CAAX and either p115 or PDZ-RhoGEF-RlucII was observed in the absence of Gα12 or Gα13 subunits in ΔGα12/13 cells (mock condition in Fig. 2b–g). The uncleavable GPR56-T383A mutant receptor exhibited a significant reduction in the constitutive activation of Gα12 when compared to the responses elicited by GPR56-WT with a maximum BRET ratio of 0,228 ± 0,06 (Fig. 2d), indicative of a lower ability of the uncleavable receptor to activate the Ga12-p115-RhoGEF pathway. When considering the PDZ-RhoGEF response, the maximal constitutive activation of Gα13 observed for GPR56-T383A was similar to the one observed for GPR56-WT (Fig. 2e). These data indicate that the uncleavable receptor maintains its constitutive activity but has a reduced ability to promote the Ga12-p115-RhoGEF while preserving a similar ability to activate Gα13-PDZ-RhoGEF. As expected, we detected no increased ebBRET signals using increasing quantities of the GPR56-Δ1–385 mutant construct (Fig. 2f, g) supporting the requirement of the ECD in constitutive GPR56 signaling.

### 10C7 stimulation of GPR56 differently activates Gα12 and Gα13

The monoclonal antibody 10C7 was generated against the GPR56 ECD as previously described [23, 30]. To assess the functional activity of 10C7 we evaluated its capacity to activate GPR56 downstream signaling. We first examined the ability of 10C7 to activate Gα12/13 proteins by monitoring the recruitment of p115-RhoGEF and PDZ-RhoGEF effectors following heterologous expression of Gα12 or Gα13, respectively, in ΔGα12/13 cells and GPR56.

**Figure 3:**
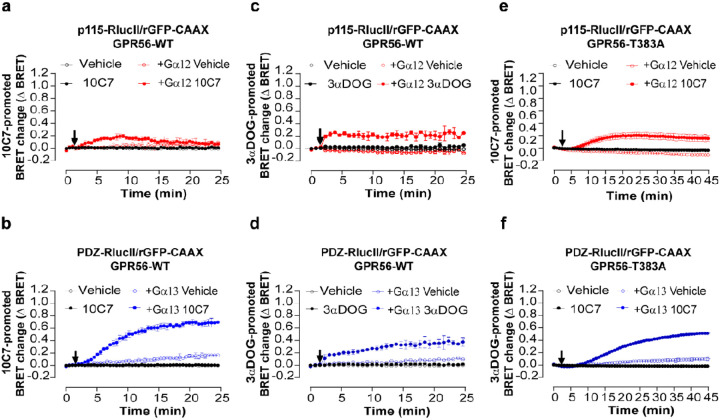
10C7 potentiates activation of Ga12/13 with a preference for Ga13 and GAIN cleavage is not necessary for 10C7 activation of GPR56-T383A. **a, b** Time course of 10C7-mediated p115-RhoGEF (a) or PDZ-RhoGEF (b) recruitment to the PM. HEK293 ΔGα12/13 cells were co-transfected with 125 ng of GPR56-WT, p115-RhoGEF-RlucII (a) or PDZ-RhoGEF-RlucII (b) and rGFP-CAAX alone or with Ga12 or Ga13 respectively, and stimulated with 20nM 10C7 or vehicle (arrow). **c**, **d** Time course of 3αDOG-mediated activation of Gα12/13 reflected by p115-RhoGEF (**c**) or PDZ-RhoGEF (**d**) recruitment to PM. HEK293 ΔGα12/13 cells co-transfected with 125 ng of WT and p115-RhoGEF-RlucII (**c**) or PDZ-RhoGEF-RlucII (**d**) and rGFP-CAAX, alone or with Gα12 or Gα13 subunits and stimulated with 10 μM of 3αDOG or vehicle (DMSO 0,1%) (arrow). **e, f** Time course of 10C7-mediated p115-RhoGEF (**e**) or PDZ-RhoGEF (**f**) recruitment to the PM. HEK293 ΔGα12/13 cells were co-transfected with 125 ng GPR56-T383A, p115 or PDZ-RhoGEF-RlucII and rGFP-CAAX alone or with Gα12 or Gα13 subunits and stimulated with 20 nM 10C7 or vehicle (arrow). Data are represented as the means ± SD of triplicate in a representative experiment that was repeated three times with similar results. Note that some control curves and error bars overlap with each other.

10C7 triggered a time-dependent and transient activation of G12 as assessed by the recruitment of p115-RhoGEF to the plasma membrane reaching a maximum ΔBRET of 0,19 ± 0,03 at 8 min of stimulation, followed by a rapid decrease, returning to a basal signal 12 min post 10C7 stimulation (Fig. 3a). Interestingly, and in contrast to G12, 10C7 promoted a stable activation of G13 as reflected by the rapid and sustained increase in ebBRET between PDZ-RhoGEF-RlucII and rGFP-CAAX reaching a maximal ΔBRET of 0,7 ± 0,01 after 24 min of stimulation (Fig. 3b). Notably, whereas the maximal increase in ebBRET observed were similar for the constitutive activation of both G protein subtypes, a larger and more sustained response was observed for Ga13 than Ga12 upon 10C7 stimulation (Fig. 3a, b). Similarly, the potency of 10C7 was greater for G13 activation with an EC50 of 4 nM whereas no saturation could be reached at the highest 10C7 concentration used for G12 (Fig. S3a, b). Taken together, these data indicate that despite the faster responses observed for Ga12 (t_1/2_ ~2,83), 10C7-stimulated GPR56 has a greater propensity to activate the G13-PDZ-RhoGEF pathway (t_1/2_ ~8). The selectivity of the 10C7-promoted responses was confirmed by the fact that 10C7 did not promote the recruitment of RhoGEF effectors in the absence of heterologously expressed Gα12 or Gα13 subunits in ΔGα12/13 cells.

When we assessed the small molecule GPR56 agonist 3αDOG [21, 27], a different activation profile was observed. First, the G12 and G13 responses promoted by 3aDOG were faster (t_1/2_ ~1,82 and ~8 for G12 and 13, respectively) than those observed with 10C7 as was mentioned above (Fig. 3c, d vs 3a, b) consistent with different mechanisms of activation. In contrast to 10C7, 3aDOG promoted sustained and stable response for both G12 and G13. Also, in contrast to what was observed with 10C7, 3aDOG induced similar response amplitudes for Ga12-p115-RhoGEF and Ga13-PDZ-RhoGEF (Fig. 3c, d). These results show that the difference in signaling of GPR56 observed toward Gα13 vs Gα12 upon stimulation with 10C7 is unique to the activation by the antibody. Such a difference in the signaling profile promoted by 3αDOG and 10C7 may not be surprising given their different binding modalities that most likely translate in different activation modes, 10C7 binding to the ECD [23, 30] whereas 3aDOG most likely binds to a binding pocket within the 7TM domains [21, 27]. In order to measure the synergy between 10C7 and 3aDOG, we perform a dual ligands stimulation of GPR56-WT (Fig S4). Concentration response curves for 3αDOG mediated recruitment of p115 or PDZ-RhoGEF effectors were carried out in the absence or presence of 10C7. For both p115 and PDZ-RhoGEF biosensors, when 10C7 was used in combination with 3αDOG, the EC50 values were the same as those measured upon 3αDOG stimulation. Together, these data could define 10C7 as a positive allosteric modulator on GPR56-WT basal activity but as a silent allosteric modulator on the 3αDOG stimulated activity that does not produce any effect on its potency.

Additionally, we were intrigued by the Gas coupling that was detected in constitutive conditions (Fig. 1c) and asked if we could detect Gs activation following 10C7 or 3aDOG stimulation. To address this question, we measured the production of cAMP after receptor stimulation, and found that cAMP was not further increased in stimulation conditions. To ensure that the Gs signal was not being lost in favor of preferential G12/13 coupling, we also used ΔGα12/13 cells and cAMP was not increased even under these conditions (Fig S5).

### 10C7 activates GPR56-T383A

We next asked whether the TA needs to be exposed for G12 and G13 activation by 10C7. As seen in Fig. 3f, the G13 activation profile observed for GPR56-T383A was very similar to that observed for GPR56-WT (Fig. 3b) upon 10C7 stimulation. In contrast, the G12 response that was transient for the WT receptor (Fig. 3a) was more sustained for GPR56-T383A (Fig. 3e). Also of note, the GPR56-T383A responses showed slower kinetics (G12: t_1/2_~10 min and G13: t_1/2_~19 min vs 2,8 min and 8 min for GPR56-WT) and reduced amplitudes towards G13 (G12: ΔBRET max: 0,2 ± 0,06 and G13: ΔBRET max: 0,5 ± 0,01 vs 0,19 ± 0,03 and 0,7 ± 0,01 for GPR56-WT) compared to GPR56-WT (Fig. 3e, f). These observations reveal that the autocleavage of the GAIN domain is not required for 10C7-mediated modulation of GPR56 signaling and that this does not necessitate an exposed TA. Yet, the lack of cleavage affects the activation dynamics especially towards G13. However, to delineate whether, regardless of TA exposure, the TA is required for 10C7-mediated modulation of the receptor, we constructed a triple mutant where in addition to the T383A mutation, we introduced two more mutations F385A and M389A to completely disrupt the TA [15, 20]. First, we verified the surface expression of GPR56 T383/F385/M389-A, showing a 2-fold increase compared to the WT (Fig S6a). We next assessed the ability of 10C7 to activate Gα12/13 in the triple mutant by measuring the recruitment of p115-RhoGEF and PDZ-RhoGEF effectors. No GPR56 T383/F385/M389-A response was detected after 30 min of stimulation with 10C7, contrary to what was observed for GPR56 T383A for p115-RhoGEF response, and GPR56 WT and T383A for PDZ-RhoGEF response (Fig S6b,c). Thus, we conclude that despite the absence of cleavage not affecting the 10C7 response, the intact GPR56 TA is also required for 10C7-mediated modulation.

### 3αDOG activates GPR56-Δ1–385 but not GPR56-T383A

**Figure 4:**
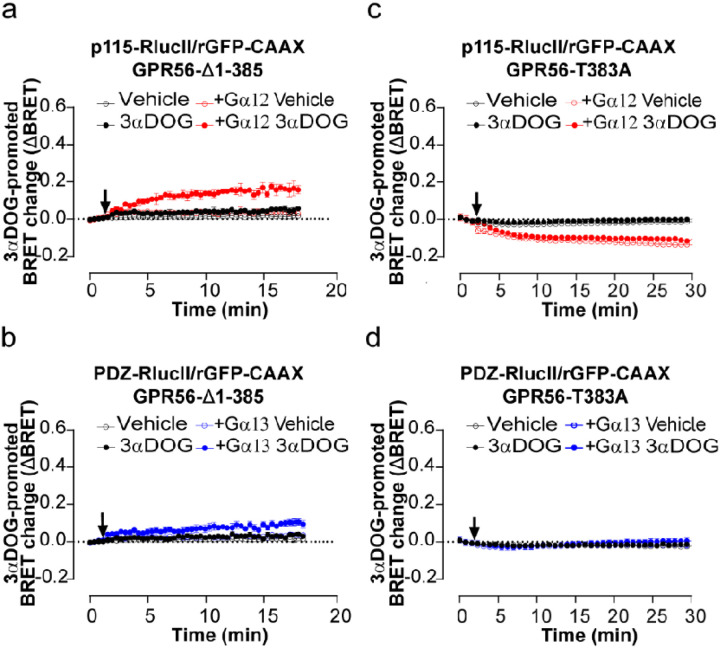
3αDOG activates GPR56-A1–385 but not GPR56-T383A. **a, c** Time course of 3αDOG-mediated activation of Ga_12/13_ proteins reflected by p115-RhoGEF or **b**, **d** PDZ-RhoGEF recruitment to the PM. HEK293T AGa12/13 cells were co-transfected with either 400 ng of Δ1–385 (**a, b**) or 125 ng of T383A (**c, d**), and p115-RhoGEF-RlucII (**a, c**) or PDZ-RhoGEF-RlucII (**b, d**) and rGFP-CAAX, alone or in presence of Gα12 or Gα13 subunits and stimulated with 10 μM of 3αDOG or vehicle (DMSO 0,1%) (arrow). Data are represented as the means ± SD of triplicate in a representative experiment that was repeated three times with similar results Note that some control curves and error bars overlap with each other.

To gain further understanding of the activation mode of G12 and G13 by 3aDOG, we investigated whether GAIN domain-mediated receptor autoproteolysis and an intact exposed TA are required for GPR56 activation. 3αDOG promoted GPR56-.Δ1–385 activation of G12 and G13 albeit to a lesser degree than that observed with GPR56-WT and, whereas the 3aDOG-promoted activation of G12 and G13 were similar for the WT-receptor or even favoring G13, the activation of G12 by GPR56-Δ1–385 was clearly more robust than G13 (Fig. 4a, b vs Fig. 3c, d). In contrast, the uncleavable T383A mutant was not activated by the synthetic ligand (Fig. 4c, d). This suggests that the uncleaved NTF may prevent the access of 3αDOG to the orthosteric site of the receptor. Altogether, these findings support a model in which 3αDOG requires the cleavage or truncation of the GPR56-ECD in order to activate both G12 and G13. They also show that an intact TA is not required for the 3aDOG-mediated activation of GPR56 and suppose a different activation mechanism from the one exhibited by the 10C7 antibody, and that distinct active conformation features of the receptor underlie preferential engagement of either G12 or G13.

**Figure 5:**
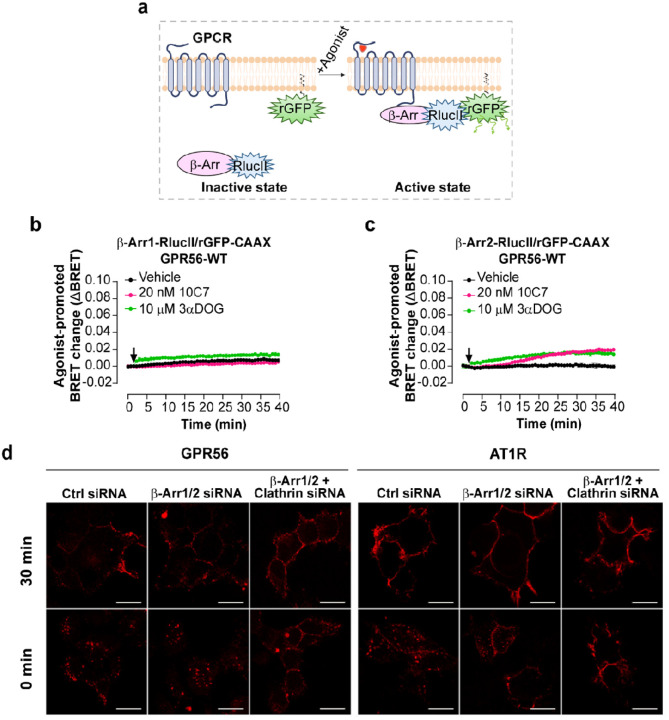
10C7 and 3αDOG trigger the recruitment of β-arrestin2 to the PM and GPR56-WT is internalized in a β-arrestin-independent but clathrin-dependent manner. **a** Schematic representation of the β-arrestin-based biosensors to monitor β-arrestin1/2 recruitment to the PM. Upon agonist stimulation, the β-Arr1/2-RlucII-tagged is recruited by the receptor to the PM inducing an increase BRET with the membrane-anchored rGFP (rGFP-CAAX), created with BioRender.com. **b,c** Time course of 10C7 and 3αDOG-mediated β-arrestin1 (b) or β-arrestin (c) recruitment to PM by GPR56-WT. HEK293 cells were co-transfected with 125 ng GPR56-WT and β-arrestin1 or β-arrestin2 -RlucII/rGFP-CAAX sensors before stimulation with 20 nM of 10C7, 10 μM of 3αDOG or Vehicle (arrow). Data are represented as the means ± SD of triplicate in a representative experiment that was repeated three times with similar results. **d** Myc-GPR56 stable HEK293 cells were treated with non-targeting siRNA (CTL-siRNA) or β-arrestin1 and 2 specific siRNA alone β-arr1/2-siRNA) or together with clathrin siRNA (Clathrin-siRNA), and also transfected with Flag-AT1R. Cell surface GPR56 and AT1R were labeled with anti-Myc polyclonal antibody and anti-Flag monoclonal antibody, respectively. To allow internalization, GPR56 and AT1R were stimulated with 10C7 (15μg/mL)) and Ang II (μM), respectively, for 30 min at 37 °C. Cells were fixed, processed for immunofluorescence and analyzed by confocal microscopy. Scale bars:10 μm.

Given that β-arrestins are well-characterized components involved in GPCR internalization [38, 39] and that a truncated form of GPR56 was described to associate with b-arrestin2 [19], we investigated whether 10C7 and 3αDOG stimulation of GPR56 promotes the recruitment of b-arrestin1 and 2. To this end, we used an ebBRET-based biosensor composed of b-arrestin1 or b-arrestin2-RlucII and rGFP-CAAX that results in an increase of the BRET signal when b-arrestins are recruited to the plasma membrane upon receptor activation (Fig. 5a). When using b-arrestin1-RlucII/rGFP-CAAX biosensor, a weak increase in the BRET signal was only observed following GPR56 stimulation with 3αDOG (Fig. 5b). However, following stimulation of GPR56 with either 10C7 or 3αDOG, a slight increase in b-arrestin2 recruitment was detected (Fig. 5c). Remarkably, the ebBRET signal increased very rapidly upon the addition of 3αDOG whereas 10 min was needed to detect an increase following the addition of 10C7; these are kinetics that are analogous to those observed for the G12/13 activation by these ligands.

We next investigated the contribution of β-arrestins to the internalization of GPR56. b-arrestin1 and b-arrestin2 were depleted in HEK293T cells stably expressing Myc-tagged GPR56 using specific siRNAs targeting b-arrestin1 and 2 (b-arrestin1/2-siRNA) or control siRNA (CTL-siRNA). Western blot analysis and quantification demonstrated that the expression levels of endogenous b-arrestin1 and 2 were reduced by 80% with the b-arrestin 1/2-siRNA when compared to control siRNA (Fig. S7a). As a functional control for the b-arrestin depletion, cells treated with the same siRNA were co-transfected with Flag-tagged angiotensin type 1a receptor (AT1R), a GPCR for which internalization was previously reported to be b-arrestin-dependent [40, 41]. Cell-surface GPR56 and AT1R were labeled with anti-myc or anti-flag antibodies, respectively, and internalization was monitored by confocal microscopy following stimulation with 10C7 for GPR56 or angiotensin II (AngII) for AT1R for 30 min at 37°C. Interestingly, 10C7-induced internalization of Myc-GPR56 was maintained in b-arrestin-depleted cells following a 30-minute incubation period (Fig. 5d, left panel). In contrast, agonist-induced Flag-AT1R internalization was markedly reduced in these cells (Fig. 5d, right panel). Of note, while Flag-AT1R was mainly located at the plasma membrane in b-arrestin depleted cells treated with agonist, a small pool of Flag-AT1R was still observed in small intracellular vesicles (Fig. 5d), probably due to the partial knockdown efficiencies (20% of b-arrestins remained after siRNA depletion). Taken together, these results indicate that the internalization of stimulated GPR56 is independent of b-arrestins.

To determine whether GPR56 internalization is clathrin-mediated, HEK293 cells stably expressing Myc-GPR56 were treated with clathrin-targeting siRNA in addition to the β-arrestin 1/2 siRNAs. qRT-PCR analysis shows that the expression levels of endogenous clathrin were reduced by 73% with siRNA clathrin compared to control siRNA (Fig. S7b). As shown in Fig. 5d, the downregulation of clathrin expression completely inhibited the internalization of both Myc-GPR56 and Flag-AT1R. Taken together, these results indicate that ligands weakly stimulated GPR56 recruitment of β-arrestin1/2 and that the receptor is internalized in a b-arrestin-independent but clathrin-dependent pathway.

### 10C7 activation of the downstream effectors of Gα12/13

**Figure 6:**
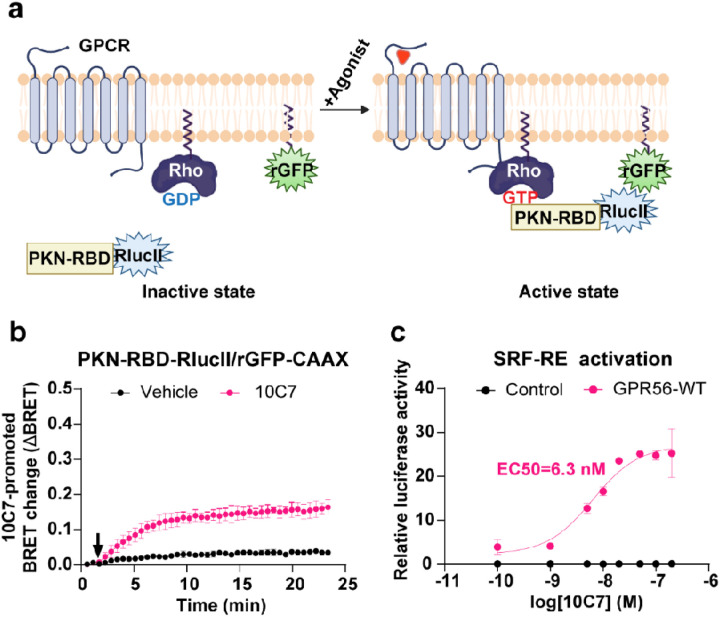
10C7 activates RhoA pathway with overexpressed GPR56-WT. **a** Schematic representation of the ebBRET-based biosensor to monitor Rho activation, created with BioRender.com. Upon agonist stimulation, the RlucII-tagged Rho-binding domain (RBD) of PKN (PKN-RBD-RlucII) recruitment to the PM increases ebBRET with the membrane-anchored rGFP (rGFP-CAAX). **b** Time course of 10C7-mediated recruitment of the RlucII-tagged PKN-RBD-RlucII to the PM. HEK293 cells were transfected with 125 ng of GPR56-WT along with PKN-RBD-RlucII and rGFP-CAAX and stimulated with the Vehicle (buffer) or 20 nM of 10C7 (arrow). For kinetics results, data are represented as the means ± SD of triplicate in a representative experiment that was repeated three times with similar results. **c** Dose-response curves of SRF-RE reporter gene activation induced by increasing concentrations of 10C7. Results are expressed as a relative luciferase activity (ratio of Firefly over Renilla luminescence). Data represent the mean of three independent experiments ± SD.

Given that 10C7 shows clear selectivity toward G13 vs G12 compared to both the constitutive activity or 3aDOG, we then assessed the downstream consequences of GPR56-mediated activation by 10C7. For this purpose, we evaluated the ability of 10C7 to activate RhoA using a Rho ebBRET sensor consisting of the Rho-binding domain (RBD) of PKN tagged to RlucII (PKN-RBD-RlucII) and rGFP-CAAX [42] (Fig. 6a). Upon Rho activation, the recruitment of PKN-RBD-RlucII to the PM increases ebBRET with the membrane-anchored rGFP-CAAX. In HEK293 cells expressing GPR56-WT and the Rho sensor, 10C7 time-dependently increased the BRET signal that reached a maximum within 10 minutes and remained stable for at least 15 min (Fig. 6b) indicating that the 10C7-promoted GPR56 activation resulted in a sustained activation of Rho. To further assess whether the 10C7-promoted activation of GPR56 could translate into gene regulation, we also used a Rho-dependant SRF-RE luciferase reporter gene assay. GPR56 stimulation by 10C7 led to a six-fold increase in SRF-RE activity with an EC50 of 6.3 nM but had no impact in cells that did not heterologously express GPR56 (Fig. 6c). Our results are in agreement with the finding of Chatterjee et al as they found that 10C7 potentiates SRF-RE activity with an EC50 of 2nM [23]

Figure 5. **10C7 and 3αDOG trigger the recruitment of β-arrestin2 to the PM and GPR56-WT is internalized in a β-arrestin-independent but clathrin-dependent manner. a** Schematic representation of the β-arrestin-based biosensors to monitor β-Arr1/2 recruitment to the PM. Upon agonist stimulation, the β-Arr1/2-RlucII-tagged is recruited by the receptor to the PM inducing an increase BRET with the membrane-anchored rGFP (rGFP-CAAX), created with BioRender.com. **b,c** Time course of 10C7 and 3αDOG-mediated β-arrestin 1 (b) or β-arrestin2 (c) recruitment to PM by GPR56-WT. HEK293 cells were co-transfected with 125 ng GPR56-WT and β-arrestin1 or β-arrestin2 -RlucII/rGFP-CAAX sensors before stimulation with 20 nM of 10C7, 10 μM of 3αDOG or Vehicle (arrow). Data are represented as the means ± SD of triplicate in a representative experiment that was repeated three times with similar results. **d** Myc-GPR56 stable HEK293 cells were treated with non-targeting siRNA (CTL-siRNA) or β-arrestin 1 and 2 specific siRNA alone (β-arr1/2-siRNA) or together with clathrin siRNA (Clathrin-siRNA), and also transfected with Flag-AT1R. Cell surface GPR56 and AT1R were labeled with anti-Myc polyclonal antibody and anti-Flag monoclonal antibody, respectively. To allow internalization, GPR56 and AT1R were stimulated with 10C7 (15μg/mL)) and Ang II (1μM), respectively, for 30 min at 37°C. Cells were fixed, processed for immunofluorescence and analyzed by confocal microscopy. Scale bars:10 μm.

### 10C7 mAb acts as a GPR56 agonist in breast cancer cells

**Figure 7:**
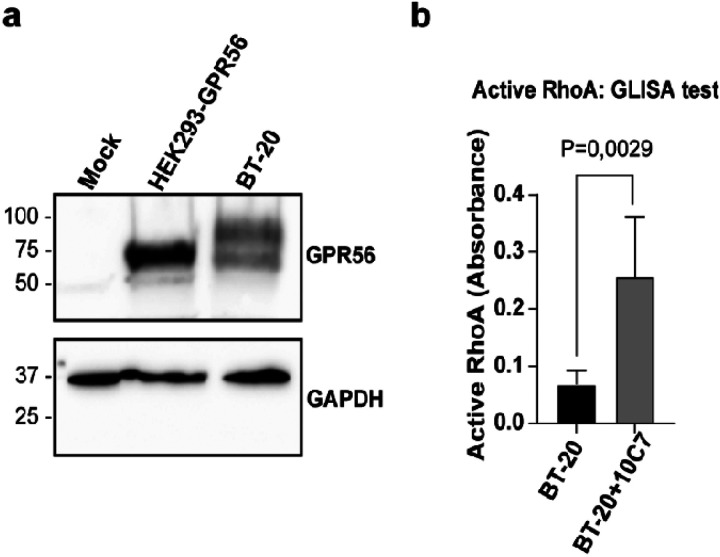
10C7 triggers RhoA activation in endogenously GPR56 expressing cells. **a** Western blotting of whole-cell lysates of HEK293 cells expressing empty vector (Mock) or GPR56-WT, and BT20 cells using 10C7 mAb. **b** RhoA ELISA test on 10C7 stimulated BT-20 cell line. BT20 cells were treated with 10C7 for 15min and active RhoA was measured by ELISA. ELISA experiment was carried out in duplicates from three independent experiments. Results are expressed as means ± SD and statistical significance analysis was performed using paired Student’s t test, *p < 0.05.

GPR56 was reported to be upregulated in breast cancer cells and to contribute to cancer cell growth and bone metastasis formation [43]. Therefore, we selected BT20 cells, a triple-negative breast cancer cell line prone to metastasis, as a model to assess whether the 10C7 could stimulate endogenously expressed GPR56. First, we verified by Western Blot the expression level of GPR56 in BT20 cells using the 10C7 mAb. As shown in Fig. 7a, GPR56 is expressed endogenously in BT20 cells. Since we detected 2 major forms of GPR56 in these cells with one band being larger than the predicted one, we verified if this increased molecular weight was due to glycosylation. Following PNGase F treatment, of HEK293 overexpressing GPR56-WT and BT20 cells, we detected band shifts for GPR56 from ~ 70 kDa to a single sharp band at around 45 kDa in both cell types, the predicted molecular weight of unglycosylated GPR56-NTF (Fig. S8) indicating that the two bands observed in BT20 cells represent differentially glycosylated forms. To investigate the effect of 10C7 on endogenously expressed GPR56, BT-20 cells were treated for 15 minutes with or without 10C7 (Fig. 7b). We found that 10C7 triggered a statistically significant 3-fold activation of RhoA. These data demonstrate that 10C7 can not only activate RhoA following heterologously expression of GPR56 in HEK293 cells but also in native endogenous GPR56 in the BT-20 breast cancer cell line.

Figure 6. **10C7 activates RhoA pathway with overexpressed GPR56-WT. a** Schematic representation of the ebBRET-based biosensor to monitor Rho activation, created with BioRender.com. Upon agonist stimulation, the RlucII-tagged Rho-binding domain (RBD) of PKN (PKN-RBD-RlucII) recruitment to the PM increases ebBRET with the membrane-anchored rGFP (rGFP-CAAX). **b** Time course of 10C7-mediated recruitment of the RlucII-tagged PKN-RBD-RlucII to the PM. HEK293 cells were transfected with 125 ng of GPR56-WT along with PKN-RBD-RlucII and rGFP-CAAX and stimulated with the Vehicle (buffer) or 20 nM of 10C7 (arrow). For kinetics results, data are represented as the means ± SD of triplicate in a representative experiment that was repeated three times with similar results. **c** Dose-response curves of SRF-RE reporter gene activation induced by increasing concentrations of 10C7. Results are expressed as a relative luciferase activity (ratio of Firefly over Renilla luminescence). Data represent the mean of three independent experiments ± SD.

## Discussion

Adhesion GPCRs (aGPCRs) represent an emerging research area since members of this GPCR family are involved in cell-cell and cell-matrix communication, crucial for cell proliferation, activation, and migration. Due to the complex structure of aGPCRs and the lack of ligands for most of these receptors, their activation modes, function and signaling profiles need to be better understood. Therefore, identifying aGPCR ligands, elucidating the molecular mechanism underlying their activity and profiling the signaling pathways engaged are of considerable interest.

The ability of selected aGPCRs to exhibit some degree of constitutive activity independently of agonists has facilitated our understanding of their signaling profiles [44–47]. Most of these studies make use of N-terminally truncated forms of the receptor to expose the TA thereby activating the receptor and enabling the measure of distal signaling events to follow their activation. In the present study, we focused on establishing the signaling profile of GPR56 and to better characterize the signaling ability of GPR56 agonists, that were until now assessed only through the monitoring of downstream reporter gene assays. We found that GPR56 directly engages both Gα12 and Gα13 and, interestingly, we show for the first time that the wild-type receptor presents weak constitutive coupling to Gαs, without leading to any measurable cAMP increase following stimulation with 10C7 or 3αDOG. GPR56 has previously been described to promote cAMP and activate PKA following stimulation with testosterone, but no evidence was provided to confirm that such activation was Gαs mediated [31]. On the other hand, others failed to observe any significant coupling to Gαs [20], this discrepancy may be explained by the cell line used, the differences on the experimental readout system or the receptor form used. While we use the WT form to assess basal constitutive activity, Stoveken et al. used receptor membranes treated with 7M urea which dissociates NTF from the CTF and exposes the TA. We next looked at how GPR56 constitutively activates G12 and G13, through the recruitment of biosensors based on the downstream signaling RhoGEF proteins p115-RhoGEF and PDZ-RhoGEF that were used to monitor G12 and G13 respectively. These biosensors allow measurement of proximal signaling following receptor activation and rapid signal detection. The constitutive recruitment of RhoGEF effectors to activated Gα12/13 may participate in the regulation of the receptor signaling and downstream effectors in physiological conditions since they serve as GTPase-activating G-protein accelerating the intrinsic rate of GTP to GDP by Gα12/13 and terminating the signaling through these subunits. Basal constitutive GPR56 activity had previously been described and assessed mainly through luciferase activation assay and GTP loading experiment in a reconstitution system and results show that GPR56 signals through the G12/13 pathway [19–21]. These previous results are in accordance with our observations as illustrated by the recruitment of specific G12/13 RhoGEF (PDZ and p115 RhoGEF).

Taking advantage of the uncleavable form of GPR56, GPR56-T383A, we show that NTF dissociation is not required to trigger constitutive GPR56-mediated G protein signaling showing a preference for G13 that reproduces the activity of the WT form. Our results are in accordance with a previous report in which the mutation of T383A did not affect SRF-RE signaling [19], this evidence shows that the T383A mutation inhibits GPR56 cleavage without affecting the activity of the TA sequence. The observed preference towards Gα13 could be attributed to a conformational rearrangement different from the WT form leading to the exposure of specific determinants resulting in such selectivity. Similar results were observed with the uncleavable form of another aGPCR, latrophilin-3, where the cleavage deficiency leads to a signalling bias [48].

In addition to GPR56’s constitutive activity, few natural ligands have been described as agonists for GPR56 [6, 24, 25] but antibodies and small molecules have been used to delineate GPR56 RhoA-SRE, SRF/RE signaling [27–29]. We show that, following GPR56 stimulation by 10C7, Gα13 is preferentially activated over Gα12. Although Gα12 and Gα13 are the most homologous among Gα subunits and share similar biochemical properties, these subunits were described to have distinct physiological functions [49, 50] and among Gα12/13 signaling GPCRs, few were found to couple solely to Gα12 or Gα13 [51, 52]. Hence favoring a preferential coupling of Gα13 over Ga12 through allosteric modulation offers the possibility of promoting a more targeted pharmacologic response in different contexts. Whether or not this could translate into physiologically or therapeutically relevant differences remains to be confirmed. In any case and despite the preferential coupling and the slow activation kinetics, 10C7 was shown to enhance RhoA-SRF activation in a Src-dependent manner through an unknown mechanism [23]. In our study, we demonstrate that stimulation of GPR56 with 10C7 enhances the recruitment of RhoGEF biosensors, which would mimic the activation of p115Rho-GEF and PDZ-Rho-GEF effectors. Our data therefore suggest that in addition to the Src-dependent mechanism that was described, RhoA could also be activated through a G12/13 dependent pathway through the activation of RhoGEFs. The precise molecular mechanism by which aGPCRs switch between active and inactive conformations still needs to be fully understood. Using an autoproteolysis-defective mutant T383A, we report that it is susceptible, as well as the WT receptor, to 10C7-mediated modulation. These results support the hypothesis that aGPCR signaling can be regulated in an autoproteolysis-independent manner where ligand binding triggers conformational changes in the GAIN or the NTF domains thereby stabilizing an active receptor conformation. On the other hand, the small molecule, partial agonist 3αDOG [21, 27], which activated both GPR56-WT and the Δ1–385 mutant, did not activate the uncleaved T383A, which confirms previous observations [21] and supports the fact that 3αDOG can activate GPR56 even with defective TA and may require an autoproteolytic cleavage between the NTF and CTF to activate GPR56. An alternative explanation is that the uncleavable form may adopt a conformational structure that prevents access of 3αDOG to the orthosteric site. Indeed,, in a recent study revealing the crystal structure of GPR56 using an autocleavage deficient mutant, it was shown that the GAIN domain was not anchored to the 7TMs keeping the TA away from the orthosteric site [15]. Furthemore, previous studies described that 3αDOG activation requires the presence of at least a part of the TA [21, 27]. Altogether, these observations support the hypothesis that 3αDOG can only activate the cleaved receptor form. This highlights a striking difference between the molecular determinants underlying GPR56 activation by 10C7 when compared to 3aDOG. Because of the slower kinetics of activation by 10C7 vs the rapid 3αDOG response, we propose that 10C7 acts as a positive allosteric modulator on GPR56-WT basal activity which operates by changing the conformation of the NTF, but as a silent allosteric modulator on the 3αDOG stimulated activity, while the smaller 3αDOG acts as an orthosteric agonist as was previously described [21, 23]. This observation may have important implications for GPR56-based therapeutic approaches.

Although the mutants used in this study provide structure-function responses, the WT form is the one to be considered on a physiological level. GPR56-WT shows a broad tissue expression in humans ranging from NK cells, β-pancreatic cells, brain cells and neural progenitor cells [5, 28, 53] and by coupling with Ga12/13, it activates SRF-RE mediated transcription in RhoA dependent manner [28]. Recently, GPR56 was reported to be the platelet collagen GPCR and activates G13 protein signaling for platelet shape change during hemostasis [53]. Furthermore, GPR56 mRNA expression was observed in many cancer tissues and was higher than their normal counterparts [10]. The exact mechanisms of GPR56 activation, signaling and implications in tumorigenesis are not completely understood. The cellular (and sub-cellular) localization of GPR56 and the coupling with G protein may vary with the cell type because of varying interacting proteins expressed in the various cells.

One of the major pathways by which GPCRs are desensitized and internalized is through β-arrestin recruitment [39]. Compared to other GPCR classes, little is known about the intracellular trafficking of aGPCRs. At least one other aGPCR, GPR64, was described to couple constitutively to β-arrestin, however only the form lacking the N-terminal fragment and not the full-length receptor was studied [54]. More recently another aGPCR, GPR125, was described to be internalized in a constitutive manner via a β-arrestin-independent pathway [55]. Understanding the mechanistic process through which GPR56 is internalized is necessary to better understand the regulation of this receptor. We show that stimulation of GPR56 with 10C7 or 3αDOG very slightly increased the recruitment of β-arrestin2. β-arrestin1 was also weakly recruited only after 3αDOG stimulation. Again, we noted faster β-arrestin2 recruitment kinetics for 3αDOG and slower β-arrestin2 recruitment kinetics for 10C7, further highlighting their distinct activation modes.

Using β-arrestin1/2 siRNA, we observed that 10C7-induced endocytosis of GPR56 is independent of β-arrestins (in contrast to AT1R for which agonist-induced internalization was strongly inhibited). This suggests that the low level of β-arrestin2 recruitment observed by BRET is not essential for GPR56 internalization. Despite the well-characterized role of arrestins in GPCR endocytosis, there is now a considerable number of examples of GPCR that do not rely on arrestins for their internalization [56]. The internalization of these GPCRs has been reported to involve various pathways including clathrin- and/or AP2-dependent pathway [57–62], caveolae-dependent pathway [63, 64], GRK-dependent pathway [65, 66], PKA-dependent [67] or Arf6-dependant pathways [68]. Using a clathrin-specific siRNA, we reveal that GPR56 internalization is clathrin-mediated after stimulation with 10C7. Endocytosis through the clathrin pathway often implicates the adaptor protein 2 (AP-2) which can interact directly with GPCRs through conserved structural motifs in intracellular loops or the C-tail and facilitate clathrin-mediated endocytosis independently of arrestins [58]. Interestingly, the C-terminal fragment of GPR56 exhibits several motifs described as conserved motifs for the interaction of AP2 with GPCR such as D^457^TSFLL^462^, a dileucine motif *[D/E]xxxL[L/l]* (x = any amino acids) [69], y^541^gpi^544^, a tyrosine motif ***YXX*** 0(0= hydrophobic amino acids) [70] and y^402^lsl^405^, a *YxxL* μ2 adaptin binding motif [71]. However, whether these specific molecular determinants are also involved in GPR56 endocytosis and interaction with AP2/clathrin remains to be determined. GPR56 therefore joins a select number of GPCRs that undergo β-arrestin-independent but clathrin-dependent internalization upon agonist stimulation.

Many pathological disorders are linked to GPR56 notably in the nervous system and cancer development. For instance, GPR56 promotes proliferation of triple-negative breast cancer cells and was found to increase breast cancer metastasis to bone[43, 72]. In our study, we show that 10C7 treatment of BT-20 cells potentiated the level of active RhoA thus demonstrating that 10C7 effectively binds endogenous GPR56 and retains function in cancer cells.

GPR56 is emerging as a therapeutic target of great interest to treat a variety of diseases ranging from neurological, hematopoietic, cancer and metabolic diseases [73]. Our findings uncover that GPR56 constitutive signaling differs from the stimulated one depending on the nature of the ligand or the structure of the receptor and deepen our understanding of the receptor’s signaling and downstream effectors recruitment that can help in the design and validation of novel small molecules and antibodies targeting this receptor.

## Data Availability

All data and analysis are included in the main text of the manuscript and the supporting material. All BRET-based biosensors described in are freely available under material transfer agreement for academic research from the Université de Montréal and can be requested to MB.
